# Early evaluation of adjuvant effects on topramezone efficacy under different temperature conditions using chlorophyll fluorescence tests

**DOI:** 10.3389/fpls.2022.920902

**Published:** 2022-07-22

**Authors:** Jinwei Zhang, Yaqiong Xie, Chunhua Zhang, Peng Zhang, Chunhong Jia, Ercheng Zhao

**Affiliations:** ^1^Institute of Plant Protection, Beijing Academy of Agriculture and Forestry Sciences, Beijing, China; ^2^MAP Field Crop Division, Sinochem Agriculture Holdings, Beijing, China; ^3^Beijing Grand Agro Chem Co., Ltd., Beijing, China

**Keywords:** topramezone, adjuvant, methylated seed oil, chlorophyll fluorescence test, early efficacy evaluation

## Abstract

Proper selection of adjuvant applications is an important strategy to enhance herbicide efficacy and reduce active ingredient input especially under adverse environmental conditions. In this study, a two-factor split-plot-design experiment was conducted to evaluate the effects of two adjuvants on the efficacy of topramezone on the grassy weed species giant foxtail (*Setaria faberi* Herrm.) and the broadleaved weed species velvetleaf (*Abutilon theophrasti* Medik.) under three different temperature conditions. The two tested adjuvants were methylated seed oil (MSO) and organosilicone. Three temperature levels, 35/30°C, 25/20°C, and 15/10°C (day/night), were used in the laboratory and greenhouse experiment. Plant chlorophyll fluorescence measurements shortly after herbicide application and classic whole-plant bioassay methods were used to evaluate the herbicide efficacy among the different treatments. Results indicated that the maximum quantum efficiency (Fv/Fm) of the top leaf of the weeds treated with topramezone mixed with MSO was significantly lower than that of the weeds treated with topramezone mixed with organosilicone and without an adjuvant at 2–3 days after treatment under all three temperature levels. The herbicide response of the plants treated with topramezone mixed with organosilicone and topramezone alone was not significantly different. These results corresponded well with the results of the classic whole-plant test. MSO has been shown to be good at enhancing the efficacy of topramezone on these weed species under all three temperature conditions. The measurement of chlorophyll fluorescence is a promising technique for evaluating the effects of adjuvants on the efficacy of herbicides shortly after herbicide treatment.

## Introduction

To enhance their efficacy, many postemergence herbicides have to be applied together with adjuvants (Hart et al., 1992; Bunting et al., 2004; Bautista et al., 2020). An adjuvant is any substance in an herbicide formulation or added to a spray tank to improve herbicidal activity or application characteristics (Foy, 1989). There are various types of adjuvants with varying degrees of effectiveness at improving herbicide efficacy. Selecting the proper adjuvant for herbicides is difficult but very important because the efficacy of herbicides on weeds is usually dependent on the herbicide type, weed species, the selected adjuvant, environmental conditions and so on (Penner, 2000). This can reduce the herbicide active ingredient input and environmental risk. Methylated seed oil (MSO) is a fatty acid from seed oil esterified with methanol. Reports have shown that MSO enhances the efficacy of several herbicides on certain weed species, as MSO contributes to increasing the penetration of herbicides into plants (Thompson et al., 1996; Young and Hart, 1998; Sharma and Singh, 2000; Pester et al., 2001; Bukun et al., 2010; Zhang et al., 2013a). Organosilicone surfactants were introduced to work as adjuvants for pesticides in the 1980s, and since then, their chemical structure and synergistic mechanism have been extensively researched (Stevens, 1993; Knoche, 1994). Because of the numerous advantages of these two adjuvants, MSO and organosilicone are typically the most commonly used adjuvants for pesticide application in China. Topramezone, a hydroxyphenylpyruvate dioxygenase inhibitor, was commercially introduced in 2006 (Grossmann and Ehrhardt, 2007) and registered in China in 2010. When applied as a postemergence herbicide, it controls a wide spectrum of annual grass and broadleaved weeds (Zhang et al., 2013a) and is safe for maize (Soltani et al., 2007; Gitsopoulos et al., 2010). In China, MSO is the only recommended adjuvant for this herbicide, as is the case in other countries. Thus, trying to find a new adjuvant for topramezone application will provide additional options for weed control in maize.

Environmental factors, such as temperature, relative humidity, soil moisture, rain, and wind, contribute to the amount and rate of herbicide uptake and the final efficacy (Zabkiewicz, 2000). In particular, the environmental temperature is variable at different latitudes or under certain small-scale regional conditions even in the same crop growing season. Temperature can influence the absorption, translocation, and metabolism of herbicide active ingredients in plants. Similarly, the effect of an adjuvant on herbicide efficacy varies under different environmental temperature conditions. One of the main functions of a right and good adjuvant is to overcome or minimize adverse factors. There has been long history on the effect of environmental conditions on the efficacy of herbicides (Hammerton, 1967; Peregoy et al., 1990; Hinz and Owen, 1994; Levene and Owen, 1995). However, as an increasingly extensive-used herbicide in maize field in China, from the Northeast to the Southwest region, there has been very few research on the impact and interaction effects of adjuvant type and environmental temperature conditions on the efficacy of this herbicide.

It is valuable and useful to evaluate the effect of an adjuvant on the efficacy of herbicides under different environmental conditions, especially under adverse conditions. A fast and nondestructive herbicide efficacy evaluation approach shortly after herbicide treatment could be an efficient method for agronomists to screen the right adjuvant for a certain herbicide. With the rapid development of plant phenotypic analysis, methods such as RGB imaging, multispectral imaging, hyperspectral imaging, thermal imaging, chlorophyll fluorescence, 3D sensing, and others have been introduced to test the response of plants under environmental (biotic and abiotic) stress efficiently (Lee et al., 2010; Belin et al., 2013; Huang et al., 2015; Lowe et al., 2017). Utilizing the improvement of these technologies, scientists in weed science also want to evaluate the efficacy of herbicides on weeds and their safety on crops (Streibig et al., 2014; Travlos et al., 2021). Chlorophyll fluorescence test has been used as a sensitive indicator of the physiological status of plants. It can monitor spatial and temporal variations by providing images of photosynthesis activity (Schreiber, 2004; Abbaspoor and Streibig, 2007; Belin et al., 2013). By utilizing this technology, Woodyard et al. (2009) evaluate the joint activity of mesotrione and atrazine in a tank-mix application on sensitive and resistant broadleaved weeds, Kaiser et al. (2013) and Wang et al. (2016, 2018) measured the herbicide resistance of *Alopecurus myosuroides* in the greenhouse and field conditions, and Li et al. (2018) identified herbicide stress in soybean shortly after treatment.

Understanding the effect of the two common used adjuvants in China in different environmental conditions, especially detecting it in a much efficient way, is beneficial to enhance herbicide efficacy and reduce active ingredient input. The objectives of this research were (a) to detect the effects of two adjuvant types (MSO and organosilicone) on the efficacy of topramezone under different environmental temperature conditions and (b) to determine whether the plant chlorophyll fluorescence test can be used as a nondestructive method to evaluate the effect of adjuvants on the efficacy of herbicides shortly after herbicide treatment.

## Materials and methods

### Chemicals and plant materials

In this study, the applied solution was prepared using a commercial herbicide and adjuvant products, including Baowei™ (336 g a.i. L^−1^ topramezone, SC, BASF Co., Ltd.), GY-HMax™ (methylated soybean oil, an MSO adjuvant, Central Research Institute of China Chemical Science and Technology), and BREAK-THRU® (S240, an organosilicone adjuvant, Omya. Agro. AG, Switzerland). The spray herbicide solutions were prepared according to the data in Table 1.

**Table 1:** Herbicide solution preparation for the experiment.

Treatment	Herbicide dose (g a.i. ha^−1^)	Adjuvant dose (%)
Topramezone alone	6.3	0
Topramezone with MSO^*^	6.3	0.300 (v/v)
Topramezone with organosilicone	6.3	0.025 (v/v)
Control	0	0

* *MSO means methylated seed oil*.

The dicotyledonous weed velvetleaf (*Abutilon theophrasti* Medic.) and monocotyledonous weed giant foxtail (*Setaria faberi* Herrm.) were selected as sample plants in this study, because they were two of the most common infested weed species in maize field in China. The weed seeds (provided by Herbiseed Co., UK) were pregerminated in plastic pots (11 × 11 × 6 cm) filled with vermiculite (2–3 cm) in a greenhouse (25/20 ± 1°C day/night, 122 μmol m^−2^ s^−1^ supplemental light for 12 h, and 55 ± 10% RH). After germination, the velvetleaf seedlings were transplanted into 11 × 11 × 12 cm plastic pots (3 plants per pot), and the giant foxtail seedlings were transplanted into 7 × 7 × 8 cm compostable pots (4 plants per pot). All the pots were filled with a mixture of vermiculite: peat: clay (1:1:1 by volume). The plants were irrigated daily with tap water. The homogeneous plants were selected as plant samples for the experiment when they had developed 3–4 true leaves.

### Experimental design and tests

The sample plants were moved into a growth chamber 2 days before herbicide application and were watered according to their demand. After 2 days of cultivation in the chamber, herbicides were applied using a track sprayer (Aro, Langenthal, Switzerland) with a spray volume of 200 l ha^−1^ (nozzle: 8002 EVS, Teejet® Spraying Systems Co., Wheaton, IL, United States) at 3.2 kPa. The sample plants were cultivated in the growth chamber for 2 more days and then moved back to the greenhouse. The plants were watered daily with tap water. The aboveground biomass of the plants was harvested 3 weeks after herbicide application and dried at 80°C for 48 h before weight measurement. The experiment was established as a two-factor split-plot design, with environmental temperature treatment in the main plots and adjuvant treatments in the subplots. Three replicates were used for each treatment, and the whole experiment was repeated once.

The temperature of the artificial growth chamber (KBF720, Binder GmbH, Tuttlingen, Germany) was set to produce a high temperature (35/30°C, day/night), moderate temperature (25/20°C, day/night) and low temperature (15/10°C, day/night). The photoperiod was adjusted to 12/12 h (day/night), and the relative humidity was adjusted to 75% for both the day and night time.

To evaluate herbicide efficacy, the PSII maximum quantum efficiency (Fv/Fm) of the fourth leaf (the top leaf of the plant), defined as Fv/Fm, was measured and recorded using a chlorophyll fluorometer (Imaging-PAM, M-Series MAXI Version, Heinz Walz GmbH, Effeltrich, Germany) at 2, 3, 4 and 5 days after treatment (DAT). The *F_v_*/*F_m_* was calculated according to equation (1):


(1) $$F_{v}/F_{m}=(F_{m}-Fo)/F_{m}$$

where Fm is the maximal fluorescence yield and F0 is the dark fluorescence yield. For the determination of F_0_, the plants were dark adapted for 30 min prior to the measurement. All measurements were conducted in a dark room under green illumination to avoid other photosynthetically active radiation except that emitted by the Imaging-PAM light source. After dark adaptation, the plants were illuminated with a light-saturated pulse of 2,634 μm m^−2^ s^−1^ photosynthetic photon flux density (PPFD) and a wavelength of 450 nm for *F_v_*/*F_M_* determination. Usually, all PSII reaction centers are open after dark adaptation, and nonphotochemical energy dissipation is minimal. During the saturation pulse, the fluorescence yield is maximal. The Imaging-PAM fluorometer also measures other parameters related to chlorophyll fluorescence, including effective quantum yield. The maximum quantum efficiency of PSII, however, was selected for this study because it remains unchanged until the next F0 and Fm determination.

While measuring the *F_v_*/*F_m_* value, chlorophyll fluorescence images were taken using a charge coupled device(CCD)camera mounted above the plant pots. The spatial resolution of the camera was 640 by 480 pixels, and the field of view was 10 by 13 cm. Only the plants were measured; the background was removed from the images. Fluorescence intensities are displayed as false colors. Light-emitting diodes (LEDs) were placed around the lens of the camera. Blue (450 nm) LED light provides pulse-modulated excitation light and simultaneously serves as actinic illumination and saturation pulses. The red long-pass filter in front of the CCD chip confined the detection window to wavelengths longer than 620 nm. In total, nine individual velvetleaf and twelve giant foxtail plants were measured for each treatment.

### Statistical analysis

To estimate the significance of the herbicide effect, the variable relative index (RI) of *F_v_*/*F_m_* and plant dry weight (DW) were calculated according to the following equations:


(2) $$RI_{F_{v}/F_{m}}=\frac{{\left(F_{v}/F_{m}\right)}_{treatment}}{{\left(F_{v}/F_{m}\right)}_{control}}$$

(3) $$RI_{DW}=\frac{{\left(DW\right)}_{treatment}}{{\left(DW\right)}_{control}}$$

The data were subjected to univariate analysis *via* the GLM process using SPSS 22.0 (version 22.0) software. The assumptions of variance analysis were tested by ensuring that the residuals were random and homogenous, with a normal distribution, using residual plots and the Shapiro–Wilk normality test. The data from two repeated experiments were combined for analysis because there were no interaction effects between the two experiments. When there was a significant interaction between the treatments of temperature and adjuvant (P<0.05), the means were separated by Fisher’s protected LSD test at the 5% level of probability.

## Results

### Effects of adjuvants as revealed by plant chlorophyll fluorescence measurements

The relative maximum quantum efficiency (*F_v_/F_m_*) index ( $RI_{F_{v}/F_{m}}$ ) of the giant foxtail treated with topramezone plus MSO under the high and moderate temperature conditions was significantly lower (*p* < 0.05) than that of the topramezone alone treatment group from 2 DAT to 5 DAT. Under low temperature conditions, similar differences between the two groups appeared after 4 DAT. In the case of topramezone applied mixed with organosilicone, there was no significant difference in the $RI_{F_{v}/F_{m}}$ value compared with that of the treatment of herbicide applied alone in any of the three temperature conditions. Additionally, the $RI_{F_{v}/F_{m}}$ value of the giant foxtail ranked as moderate < high < low for each adjuvant treatment from 3 DAT to 5 DAT (Table 2).

**Table 2:** Effects of two adjuvants on the maximum quantum efficiency (*F*_v_/*F*_m_) of giant foxtail (*Setaria faberi* Herrm.) at 2–5 days after treatment (DAT).

Days After Treatment (DAT)	Adjuvant	${\mathrm{RI}}_{F_{v}/F_{m}}$ $RI_{F_{V}}{/}_{F_{m}}$		
		High temperature	Moderate temperature	Low temperature
2	Alone	0.97 ± 0.07aA	0.98 ± 0.04aA	0.99 ± 0.02aA
	Org	0.89 ± 0.13abA	0.95 ± 0.06abA	1.00 ± 0.02aA
	MSO	0.89 ± 0.20bB	0.86 ± 0.08bB	0.99 ± 0.02aA
3	Alone	0.79 ± 0.25aB	0.57 ± 0.19abC	0.96 ± 0.03aA
	Org	0.78 ± 0.20aB	0.60 ± 0.19aC	0.98 ± 0.04aA
	MSO	0.73 ± 0.19aB	0.55 ± 0.16bC	0.96 ± 0.05aA
4	Alone	0.56 ± 0.26aB	0.32 ± 0.09aC	0.68 ± 0.17aA
	Org	0.42 ± 0.21abB	0.36 ± 0.12aC	0.75 ± 0.20aA
	MSO	0.37 ± 0.22bA	0.26 ± 0.07bC	0.54 ± 0.09bA
5	Alone	0.45 ± 0.23aB	0.28 ± 0.08aC	0.49 ± 0.16aA
	Org	0.36 ± 0.17aB	0.23 ± 0.09aC	0.57 ± 0.27aA
	MSO	0.27 ± 0.08bB	0.13 ± 0.04bC	0.39 ± 0.15bA

*The topramezone dose was 6.3 g a.i. ha^−1^; control means treated with tap water; Alone means that topramezone was applied alone; Org means that topramezone was applied with a tank-mix of organosilicone; MSO means topramezone was applied with a tank-mix of MSO. The means in the same column followed by a common letter are not significantly different at p = 0.05 (vertical comparison). The means in the same row followed by a capital common letter are not significantly different at p = 0.05 (horizontal comparison)*.

**Figure 1:**
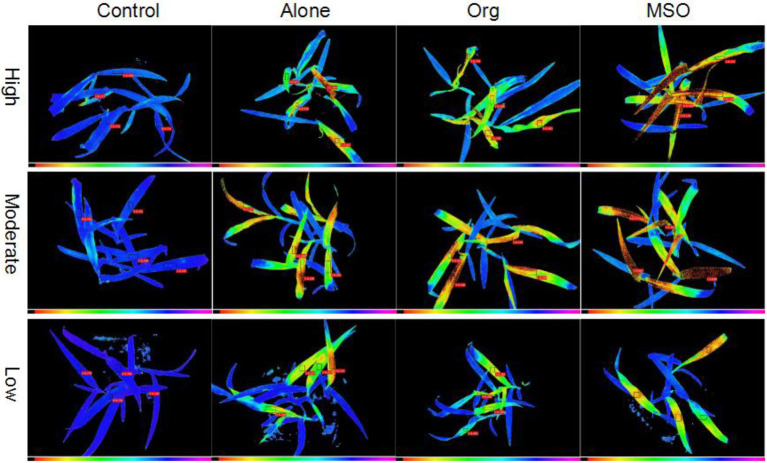
Chlorophyll fluorescence images of giant foxtail at 5 days after treatment. The topramezone dose was 6.3 g a.i. ha^−1^; control means treated with tap water; Alone means topramezone was applied alone; Org means topramezone was applied with a tank-mix of organosilicone; MSO means topramezone was applied with a tank-mix of MSO.

The chlorophyll fluorescence images taken at 5 DAT showed that the weed treated with herbicide mixed with MSO was injured more severely than the weeds treated with herbicide alone and mixed with organosilicone under all 3 temperature conditions (the false color of the normal plant leaves was blue, while the false color of the leaves changing from green to yellow and even to black demonstrated that the plants were injured more severely). In addition, the plants treated under high and moderate temperature conditions were injured more severely than those treated under low temperature conditions for each herbicide treatment (Figure 1).

**Figure 2:**
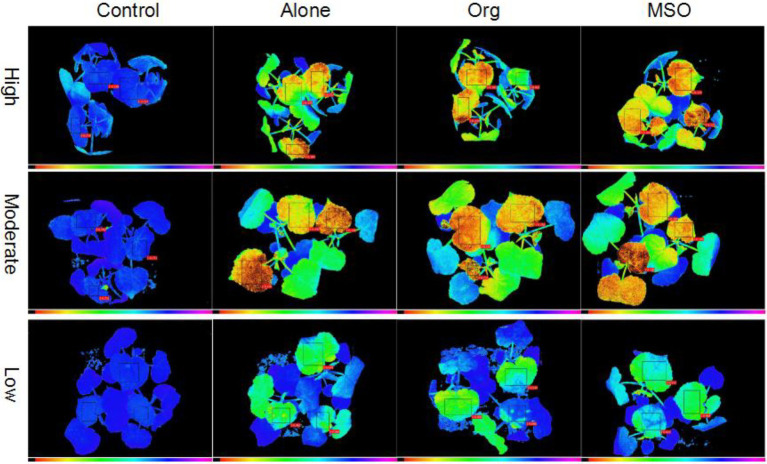
Chlorophyll fluorescence images of velvetleaf at 3 days after treatment. The topramezone dose was 6.3 g a.i. ha^−1^; control means treated with tap water; Alone means topramezone was applied alone; Org means topramezone was applied together with a tank-mix of organosilicone; MSO means topramezone was applied together with a tank-mix of MSO.

Similar to the case of giant foxtail, the $RI_{F_{v}/F_{m}}$ value of velvetleaf treated with topramezone together with a tank-mix of MSO under the high and moderate temperature conditions was significantly lower (*p* < 0.05) than that of the topramezone alone treatment group from 2 DAT to 5 DAT, while there were no differences under the low temperature conditions. With respect to the treatment of topramezone together with a tank-mix of organosilicone, there was no significant difference compared with the treatment of topramezone alone under any of the three temperature conditions. With respect to all three herbicide treatments, the $RI_{F_{v}/F_{m}}$ value of the treated velvetleaf under moderate and high temperature conditions was significantly lower (*p* < 0.05) than that under low temperature conditions (horizontal comparison). The chlorophyll fluorescence images taken at 5 DAT also showed that velvetleaf plants treated with herbicide together with a tank-mix of MSO were more injured than those treated with herbicide alone and together with organosilicone under moderate and high temperature conditions, while the difference was not apparent under low temperature conditions (Figure 2).

The abovementioned results indicated that the MSO adjuvant significantly enhanced the efficacy of topramezone under all temperature conditions for giant foxtail and under high and moderate temperature conditions for velvetleaf, while the effect of organosilicone on enhancing the efficacy was not significant for either weed species. Additionally, the efficacy of topramezone was better under relatively high temperatures than under relatively low temperature conditions for giant foxtail and velvetleaf after it was tank-mixed with MSO (Table 3).

**Table 3:** Effects of two adjuvants on the maximum quantum efficiency (*F*_v_/*F*_m_) of velvetleaf (*Abutilon theophrasti* Medik.) at 2–5 days after treatment (DAT).

Days After Treatment (DAT)	Adjuvant	${\mathrm{RI}}_{F_{v}/F_{m}}$ $RI_{F_{V}}{/}_{F_{m}}$		
		High temperature	Moderate temperature	Low temperature
2	Alone	0.92 ± 0.07aB	0.92 ± 0.07aB	0.99 ± 0.03aA
	Org	0.88 ± 0.10abA	0.88 ± 0.08abA	1.00 ± 0.03aA
	MSO	0.86 ± 0.09bB	0.84 ± 0.08bB	0.99 ± 0.03aA
3	Alone	0.72 ± 0.15aB	0.69 ± 0.14aB	0.87 ± 0.20aA
	Org	0.72 ± 0.16aB	0.63 ± 0.17abB	0.90 ± 0.15aA
	MSO	0.59 ± 0.09bB	0.54 ± 0.12bB	0.86 ± 0.23aA
4	Alone	0.58 ± 0.19aB	0.52 ± 0.20aB	0.79 ± 0.17aA
	Org	0.45 ± 0.13abB	0.47 ± 0.24abB	0.83 ± 0.15aA
	MSO	0.39 ± 0.10bB	0.38 ± 0.16bB	0.78 ± 0.19aA
5	Alone	0.46 ± 0.11aB	0.48 ± 0.18aB	0.70 ± 0.12aA
	Org	0.34 ± 0.12abB	0.39 ± 0.31abB	0.67 ± 0.19aA
	MSO	0.25 ± 0.09bB	0.32 ± 0.21bB	0.69 ± 0.08aA

*The topramezone dose was 6.3 g a.i. ha^−1^; control means treated with tap water; Alone means that topramezone was applied alone; Org means that topramezone was applied together with a tank mix of organosilicone; MSO means topramezone was applied together with a tank mix of MSO. The means in the same column followed by a common letter are not significantly different at P = 0.05 (vertical comparison). The means in the same row followed by a capital common letter are not significantly different at p = 0.05 (horizontal comparison)*.

### Effects of adjuvants according to whole-plant biomass measurements

The relative dry weight index (*RI*_DW_) of both weed species treated with topramezone together with MSO was significantly lower (*p* < 0.05) than that treated with topramezone applied alone and together with organosilicone under all three temperature conditions, while the difference between the last two treatments was not significant under any of the three temperature conditions at 3 weeks after treatment (WAT). Additionally, the *RI*_DW_ value under the different temperature conditions ranked as high < moderate < low for both weed species for each adjuvant treatment (Table 4).

**Table 4:** Effects of two adjuvants on the dry weight of giant foxtail (*Setaria faberi* Herrm.) and velvetleaf (*Abutilon theophrasti* Medik.) at 3 weeks after application.

Weed	Adjuvant	*RI*_DW_		
		High temperature	Moderate temperature	Low temperature
Giant foxtail	Alone	0.36 ± 0.08aB	0.38 ± 0.09aAB	0.51 ± 0.19aA
	Organosilicone	0.29 ± 0.14abB	0.41 ± 0.20aAB	0.46 ± 0.18abA
	MSO	0.21 ± 0.08bB	0.32 ± 0.06bAB	0.40 ± 0.08bA
Velvetleaf	Alone	0.41 ± 0.22aB	0.46 ± 0.30aB	0.66 ± 0.37aA
	Organosilicone	0.38 ± 0.23aB	0.38 ± 0.30aB	0.59 ± 0.38abA
	MSO	0.23 ± 0.04bB	0.25 ± 0.07bB	0.48 ± 0.20bA

*The topramezone dose was 6.3 g a.i. ha^−1^; control means treated with tap water; Alone means that topramezone was applied alone; Org means that topramezone was applied together with a tank mix of organosilicone; MSO means topramezone was applied together with a tank mix of MSO. The means in the same column followed by a common letter are not significantly different at P = 0.05 (vertical comparison). The means in the same row followed by a capital common letter are not significantly different at p = 0.05 (horizontal comparison)*.

**Figure 3:**
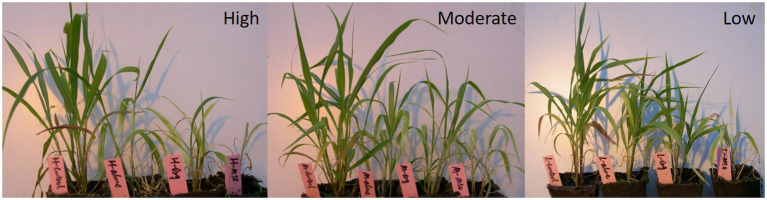
Images of giant foxtail taken at 3 weeks after herbicide application. The topramezone dose was 6.3 g a.i. ha^−1^; control means treated with tap water; Alone means topramezone was applied alone; Org means topramezone was applied together with organosilicone; MSO means topramezone was applied together with MSO.

**Figure 4:**
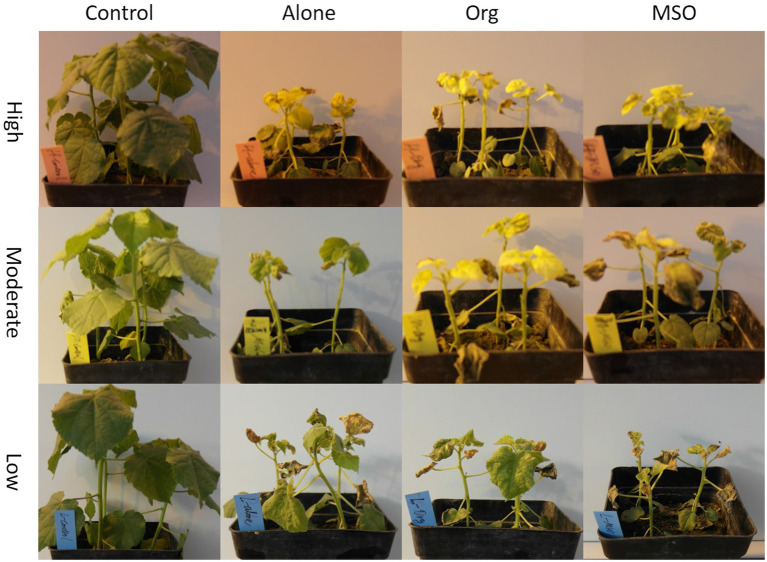
Images of velvetleaf taken at 3 weeks after application. The topramezone dose was 6.3 g a.i. ha^−1^; control means treated with tap water; Alone means topramezone was applied alone; Org means topramezone was applied together with organosilicone; MSO means topramezone was applied together with MSO.

The images taken at 3 WAT also apparently showed that both weed species treated with topramezone together with MSO were injured more than those treated with the other adjuvant and applied alone under all temperature conditions (Figures 3, 4). The results demonstrated that the MSO adjuvant significantly but not the organosilicone adjuvant enhanced the efficacy of topramezone under all temperature conditions for both weed species and that the efficacy of topramezone was better under relatively high temperature conditions than under relatively low temperature conditions.

## Discussion

### Effects of two adjuvants on enhancing the efficacy of topramezone under different environmental temperatures

In our study, both the leaf chlorophyll fluorescence measurements and whole-plant bioassay results demonstrated that the MSO adjuvant significantly enhanced the efficacy of topramezone under all temperature conditions for both weed species, especially for those under relatively high temperatures. Zollinger (2010) summarized that the MSO adjuvant had the unique advantage of enhancing herbicide efficacy when applied at reduced rates under adverse environmental conditions (e.g., hot weather, low relative humidity, and high temperature). In our case, when the plants were cultivated in the 35/30°C (day/night) conditions (the high temperature conditions), the dose of herbicide we applied (6.3 g a.i. ha^−1^) was only 1/4 of the recommended dose (the recommended dose of topramezone registered in China is 22.5–27.0 g a.i. ha^−1^). Therefore, our result is mostly consistent with the results in the report of Zollinger. Our previous research (Zhang et al., 2013b) showed that MSO enhanced the efficacy of topramezone on giant foxtail and velvetleaf by decreasing the solution surface tension and leaf-droplet contact angle and by increasing both the spread area and wetting time on weed leaf surfaces. This resulted in a decreased crystal amount of the active ingredient and an increased foliar uptake and final translocation of the active ingredient in the plants. Additionally, studies have shown that the absorption and translocation of herbicide active ingredients in plants decreased under high temperature stress, which ultimately decreased herbicide efficacy (Hawxby et al., 1972; Devine et al., 1983; Coetzer et al., 2001). Thus, the application of MSO adjuvants could contribute to the enhancement of herbicide efficacy, especially under adverse environmental conditions.

Adjuvant organosilicone had no effect on the efficacy of topramezone under any of the three temperature conditions. Organosilicone adjuvants usually enhance the efficacy of certain herbicides by reducing the surface tension of the spray solution, promoting infiltration of the active ingredient into stomata, and increasing droplet spreading over the leaf surface (Field et al., 1992). Though a large number of studies have demonstrated good effects of organosilicone on enhancing the efficacy of herbicides with many different modes of action, there are still some reports indicating antagonistic action between L-77 (a type of organosilicone adjuvant) and glyphosate (Sharma and Singh, 2000). This is similar to the findings in our study; hence, the reason (perhaps from the perspective of deposition, retention, uptake, translocation and so on) needs to be further studied in future.

### Chlorophyll fluorescence measurement as a method to evaluate the effect of adjuvants on herbicide

Measuring changes in the chlorophyll fluorescence induction curve (Kautsky curve) has been used in plant photosynthesis research (Christensen et al., 2003; Korres et al., 2003). This method is effective at providing a snapshot of the physiological status of a plant exposed to various stress factors and contains important information about the photosynthetic apparatus. Because of its nondestructive, highly sensitive, rapid speed and easy-to-operate characteristics, this method has been used to measure the effects of herbicides that inhibit photosystem II and those with other modes of action (Habash et al., 1985; Percival et al., 1992; Klem et al., 2002). With the development of this technology and new instruments, Wang et al. (2018) demonstrated that chlorophyll fluorescence can be used to identify the effects of ALS (acetolactate synthase) and ACCase (acetyl CoA carboxylase) inhibitor herbicides on the PSII of weed species and crops under different growing conditions. Similar to other 4-hydroxyphenylpyruvate dioxygenase (4-HPPD) inhibitors, topramezone blocks the formation of homogentisate by inhibiting 4-HPPD (Grossmann and Ehrhardt, 2007). As homogentisic acid is a precursor of the most common plastoquinone (PQ-9), the electron transport efficiency between PSI and PSII decreases after the inhibition of HPPD (Xu et al., 2019), and the photosynthesis of herbicide-treated plants becomes interrupted. Thus, less energy can be used by the plants *via* photosynthesis and is therefore reemitted as chlorophyll fluorescence in a shorter wavelength compared with that which occurs in unstressed status. Therefore, chlorophyll fluorescence imaging technology should theoretically be capable of evaluating the efficacy of such mode of herbicides.

In our case, we employed chlorophyll fluorescence imaging technology to evaluate the effects of adjuvants on herbicide efficacy under different environmental temperature conditions. The classic whole-plant bioassay and plant chlorophyll fluorescence measurement results at 2-5 DAT under high and moderate environmental temperatures corresponded well with each other for both the grassy weed giant foxtail and the broadleaved weed velvetleaf. This is quite similar to the result of Vranjes et al. (2019) when they did their research on the response of *chenopodium album* and *abutilon theophrasti* to the treatment of mesotrione. The $RI_{F_{v}/F_{m}}$ value of the treatment involving the herbicide applied together with MSO in a tank mixture was significantly decreased compared with that of the treatment involving the herbicide applied alone. In the case of low temperature, the chlorophyll fluorescence measurement at 2-5 DAT was not consistent for velvetleaf, and the $RI_{F_{v}/F_{m}}$ value did not significantly vary among the different adjuvant treatments. Hence, chlorophyll fluorescence measurements are capable of evaluating the effects of adjuvants on the efficacy of herbicides under relatively high environmental temperature conditions for some grassy weed species, but attention should be paid under relatively low temperature conditions and for some broadleaved weed species. As stated above, this technology has already been applied for herbicide efficacy evaluation in the field accompanying the improvement of technology and new instruments (Wang et al., 2016, 2018; Li et al., 2018). Hence this method will accelerate the progress of screening the right adjuvant for herbicides and improve the digital component of classic herbicide bioassays practically.

## Conclusion

Selecting an appropriate spray adjuvant for herbicides under different environmental conditions is an important strategy to enhance the efficacy of herbicides, reduce the application dose, and enhance environmental safety. Both the weed leaf chlorophyll fluorescence test and the whole-plant bioassay results demonstrated that the MSO adjuvant significantly enhanced the efficacy of topramezone under all temperature conditions for both weeds, the grassy weed species giant foxtail (*Setaria faberi* Herrm.) and the broadleaved weed species velvetleaf (*Abutilon theophrasti* Medik.), especially under relatively high temperature conditions. However, the organosilicone adjuvant had no effect on the efficacy of the herbicide on either weed species under any of the temperature conditions. The underlying reason (perhaps from the aspect of deposition, retention, uptake, translocation and so on) needs to be further studied. There was a relatively good correlation between chlorophyll fluorescence measurements and whole-plant bioassay results for both weed species under high and moderate temperature conditions. Hence, chlorophyll fluorescence measurements should be capable of evaluating the effects of adjuvants on herbicide efficacy under certain environmental conditions. However, attention should still be paid under relatively low temperature conditions for broadleaved weed species.

## Data availability statement

The raw data supporting the conclusions of this article will be made available by the authors, without undue reservation.

## Author contributions

JZ contributed to conceptualization, methodology, data curation, and manuscript writing. YX contributed to investigation, validation and resources. CZ helped in investigation and resources. PZ contributed to investigation and resources. CJ contributed to supervision and funding acquisition. EZ helped in supervision, validation and resources. All authors contributed to the article and approved the submitted version.

## Funding

This research was supported by the China National Key Research and Development Project (No. 2017YFD0201808).

## Conflict of interest

JZ was employed by Institute of Plant Protection, Beijing Academy of Agriculture and Forestry Sciences. YX employed by MAP Field Crop Division, Sinochem Agriculture Holdings. CZ and PZ were employed by Beijing Grand Agro Chem Co., Ltd.

The remaining authors declare that the research was conducted in the absence of any commercial or financial relationships that could be construed as a potential conflict of interest.

## Publisher’s note

All claims expressed in this article are solely those of the authors and do not necessarily represent those of their affiliated organizations, or those of the publisher, the editors and the reviewers. Any product that may be evaluated in this article, or claim that may be made by its manufacturer, is not guaranteed or endorsed by the publisher.
